# Potential for a commercial inland fishery or just another water storage facility at Spring Grove Dam, KwaZulu-Natal, South Africa?

**DOI:** 10.2989/16085914.2024.2373096

**Published:** 2024-10-05

**Authors:** Matthew J Burnett, Céline Hanzen, Alex Whitehead, Gordon C O'Brien, Colleen T Downs

**Affiliations:** 1 Centre for Functional Biodiversity, School of Life Sciences, University of KwaZulu-Natal, Pietermaritzburg, South Africa; 2 Institute of Natural Resources NPC, Pietermaritzburg, South Africa; 3 School of Biology and Environmental Sciences, Faculty of Agriculture and Natural Sciences, University of Mpumalanga, Nelspruit, South Africa; 4 Gulbali Institute, Charles Sturt University, Albury, New South Wales, Australia

**Keywords:** fish diversity, impoundments, invasive species, *Labeobarbus natalensis*, *Micropterus*, small-scale fisheries, trout

## Abstract

South Africa's inland fisheries are generally undervalued, though there is developing interest in the sector. Spring Grove Dam in the KwaZulu-Natal Midlands became operational as a water storage facility in 2013 to provide water to the growing urban population in the province. We assessed whether the impoundment could potentially support commercial fisheries as had been proposed during its design and construction. We conducted seasonal fish community surveys from 2020 to 2021 at sites along the impoundment and in the mainstem of the Mooi River which feeds into the impoundment. We recorded a total of nine fish species, including six non-native fishes with invasive characteristics. Only three of 10 expected native species were found, namely the KwaZulu-Natal yellowfish *Labeobarbus natalensis*, chubbyhead barb *Enteromius anoplus* and African longfin eel *Anguilla mossambica*. Catch per unit effort (CPUE) was low in the sampling surveys using mostly gillnets and fyke nets, and relatively low yields (14.97 tonnes yr^−1^) were subsequently calculated for the impoundment, making commercial fisheries unviable. However, the existing subsistence and recreational fisheries for non-native salmonids and the native *L. natalensis* would benefit by controlling the distribution range and abundances of non-native centrachids *Micropterus* spp. We offer recommendations and suggestions for management plans for Spring Grove Dam in the context of local fish diversity, a changing climate, further construction of water storage facilities on east-flowing rivers in the province, and a nationally neglected inland fishery industry.

## Introduction

African inland fisheries are considered the second largest by catch per tonnage globally (Funge-Smith 2018). They contribute an estimated 26% (~6.3 U$ billion) of the total African fisheries’ gross domestic product (De Graaf and Garibaldi 2015; Chan et al. 2019). This translates to over 12.3 million people employed in the African inland fisheries sector (representing ~2% of the African population). A further 5 million people are directly linked with the fisheries value chain (e.g. via processing, preservation, transport, marketing, production, and maintenance of boats and gear) (Tveldten et al. 1996; Welcomme 2011; Youn et al. 2014; De Graaf and Garibaldi 2015). However, many African inland fisheries go unnoticed despite their role in alleviating poverty and achieving Sustainable Development Goals (SDGs) (Lynch et al. 2017, 2019). For example, South African fisheries are ranked 6th among nine southern African countries (including Zambia and Angola). Yet, no catch reports have been submitted to the Food and Agriculture Organisation (FAO) since 1990, and subsequent estimates highlight the neglect of inland fisheries on a national level (Britz et al. 2015; Funge-Smith 2018).

The policies and regulations around South African inland fisheries are complex and have been extensively discussed in the literature (e.g. Ellender et al. 2014; Hara and Backeberg 2014; Britz 2015; Britz et al. 2015; Camp et al. 2020). These include complexities around regulations, national laws, non-native species management and access to water bodies (Ellender et al. 2010, 2014; Britz et al. 2015). Historically, the South African government preferred promoting non-native fishes over native fishes for inland fisheries (Harrison et al. 1963; McCafferty et al. 2012; Weyl et al. 2018). For instance, after several attempts to establish non-native trout, a recreational salmonid fishery was established in the 1890s, which since has achieved a relatively large economy (Du Preez and Lee 2010; Weyl et al. 2018).

The National Environmental Management: Biodiversity Act 10 of 2004 (NEM:BA) (RSA 2004) prioritises the conservation of native biodiversity and recognises several non-native fish species as invasive, including economically important fishery species such as black bass *Micropterus* spp. and carp *Cyprinus* spp. (Ellender and Weyl 2014; Woodford et al. 2017; DEFF 2020). The implementation of NEM:BA was intended to enforce the protection and enhancement of South Africa’s rich native biodiversity threatened by invasive species (RSA 2004). The inclusion of salmonids on the NEM:BA Alien and Invasive Species List (DEFF 2020) was controversial, largely because of lobbying from angling clubs that view the Act as a threat to the associated economic value chain, which includes the practice of wild stocking from hatchery-reared fish (Woodford et al. 2017). An important question to consider is how to manage native freshwater biodiversity, especially fish diversity. This is in the context of non-native-based fisheries having relatively high economic value despite their potential to negatively affect native biodiversity in inland aquatic ecosystems as well as the demarcation of South Africa’s National Freshwater Ecosystem Priority Areas (NFEPA) (Weyl et al. 2007, 2021; Nel et al. 2011; Britz et al. 2015).

Inland fisheries in South Africa comprise three main sectors: commercial, small-scale (Tapela et al. 2015) and recreational (McCafferty et al. 2012). Subsistence fishing is not explicitly defined (DFFE 2021), and sport fishing is included in recreational fisheries (Potts et al. 2022). Recreational fishing (e.g. flyfishing, bank angling, bait and art-lure fishers) contributes significantly to the economy of South Africa, more than inland commercial fisheries (Britz et al. 2015; Potts et al. 2022). For example, the yellowfish *Labeobarbus* spp. angling industry on the Vaal River accounts for USD 19 million (ZAR 133 million) per season (Brand et al. 2009), and the salmonid industry generates USD 1.8 million (ZAR 13.5 million) per season in the Eastern Cape Province (Du Preez and Lee 2010). Little economic data are available for other fisheries and targeted species across the country, including commercial fishery ventures. However, the number of recreational fishers is conservatively estimated to represent only 5% of the angling population, including the informal sector (Britz et al. 2015), with ~7 427 registered recreational fishers across four main angling clubs in South Africa (Britz et al. 2015). Most of these fishers use impoundments assigned to their angling club’s particular interests (Barkhuizen et al. 2017; Potts et al. 2022). Small-scale fishers (hereafter ‘subsistence fishers’) comprise some informal sectors and are largely unaccounted for in South Africa (Funge-Smith 2018; Weyl et al. 2021).

In South Africa, the majority of inland fisheries (>3 150) have been artificially created in water storage facilities constructed since the 1960s, altering natural freshwater ecosystems (McCafferty et al. 2012; Funge-Smith 2018). Despite this, one socio-economic trade-off when developing water storage facilities is the potential for developing commercial fisheries in the impoundments created (Hara and Backeberg 2014; Jackson et al. 2016a; Funge-Smith 2018). Literature on commercial fisheries in impoundments in South Africa is scarce, although the general failure of these inland fisheries has been highlighted (McCafferty et al. 2012; Britz et al. 2015; Barkhuizen et al. 2016; Weyl et al. 2021). Failures can be attributed to a history of limited access to resources, low demand for freshwater fish, lack of inland fisheries policies, unclear fisheries objectives, and reduced yields over time (McCafferty et al. 2012; Barkhuizen et al. 2016; Weyl et al. 2017). Regardless, more impoundments are planned for construction to meet increasing water demands in the region, and the potential fisheries associated with them are still seen as a trade-off (Jackson et al. 2016a; Bester et al. 2019).

**Figure 1:**
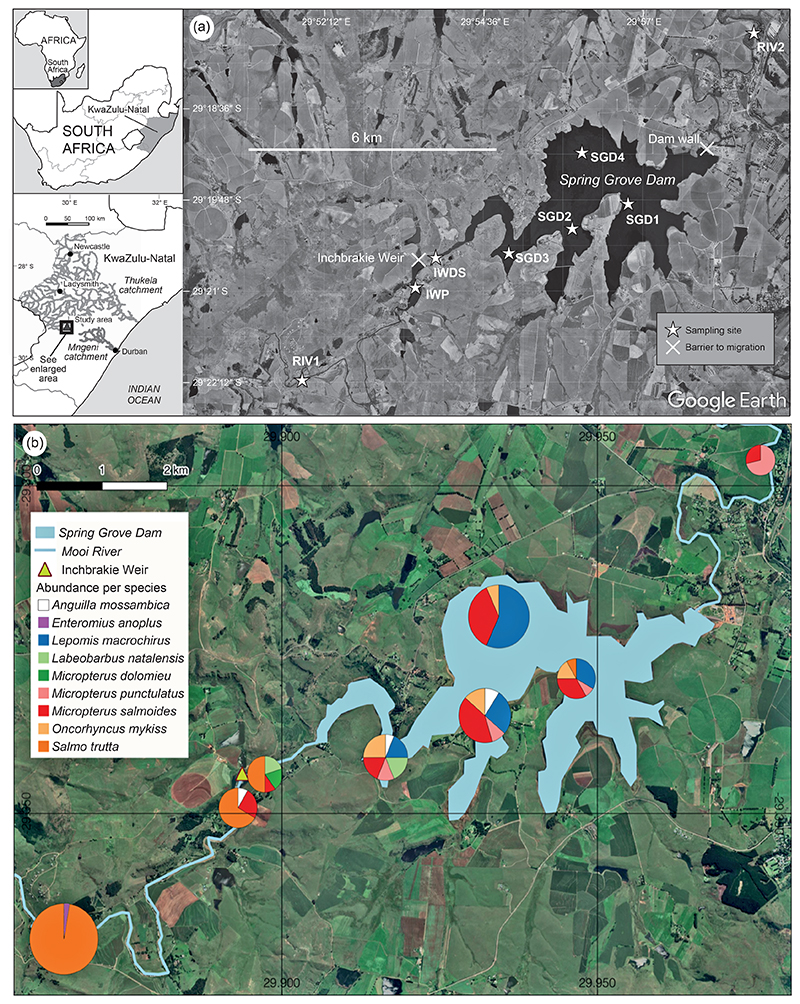
(a) Map of the study area and sampling sites on Spring Grove Dam and the Mooi River, KwaZulu-Natal, South Africa; (b) map showing the relative abundances of fish species caught at the sampling sites (see section ‘Sampling’ for abbreviations)

Spring Grove Dam was constructed in the KwaZulu-Natal (KZN) Midlands (Figure 1) to transfer water from the Thukela catchment to the Mngeni catchment through the Mooi–Mgeni Transfer Scheme, and thereby increase the water supply to over 4 million people in the Msunduzi and eThekwini municipalities (Nemai 2006). It became operational as a water storage facility in 2013. Construction of the dam was controversial since it would likely alter the associated trout fishery in the upper Mooi River by inundating a natural barrier and altering the river habitat (Nemai 2006; TCTA 2011, 2012). That natural barrier constituted a waterfall, the Inchbrakie Falls, which kept the invasive smallmouth bass *Micropterus dolomieu* from migrating upstream into the upper Mooi River and negatively impacting the established recreational fishery for the non-native brown trout *Salmo trutta* (Nemai 2006; TCTA 2011). However, since completion of the dam, policies for managing these waters and the fish resources are unclear and have been disputed by private stakeholders and governing authorities (Hara and Backeberg 2014; Britz et al. 2015; Woodford et al. 2017).

Construction of Spring Grove Dam as a water storage facility changed the landscape, which has impacted economically important river-based fisheries. This situation merited an evaluation of the impoundment in terms of its commercial fisheries potential. Thus, this study aimed to (i) assess the fish community in Spring Grove Dam, and (ii) evaluate the impoundment’s potential for supporting inland commercial fisheries. Based on our findings, we suggest inland fisheries management plans for the impoundment and surrounding river. We also include an evaluation of the native fishes that were expected versus the species recorded in the study area to underscore the potential threat of non-native fishes to native fishes, notwithstanding the inclusion of the latter into potential commercial fisheries. Thus, this study was undertaken not only from a commercial fisheries perspective but also from a fish diversity perspective, to holistically assess the impact of Spring Grove Dam on the receiving environment, similar to the study objectives of Longin et al. (2021) and Nogueira et al. (2021).

## Materials and methods

### Study area

The study area is in the upper Thukela catchment (Figure 1), the largest catchment in KZN and the second largest in the country, covering ~30 000 km^2^ (DWAF 2002). The Mooi River runs for ~260 km from its source in the Drakensberg Mountains to its confluence with the mainstem of the Thukela River, north of Greytown. The scope of the study included the upper reaches of the Mooi River, covering ~40 km from Invermooi Estate to the town of Rosetta, including Spring Grove Dam and the Inchbrakie Fish Barrier Weir (hereafter ‘Inchbrakie Weir’) (Figure 1). Spring Grove Dam captures water from a catchment area of ~344 km^2^ and holds ~139.5 million m^3^ of water when full. The dam wall is 37.5 m high and 607 m wide, and the impoundment has a surface area of 1 021.8 ha at full capacity (TCTA 2012). Construction of the dam inundated a natural fish barrier, the Inchbrakie Falls, which is still exposed when the water level drops below 60%. Consequently, the Inchbrakie Weir (Figure 1) was built as a fish barrier, mainly against the invasive smallmouth bass *M. dolomieu*. It consists of an instream weir with a 7-m wall, and its impoundment holds 150 000 m^3^ of water. Above the inlet to the Inchbrakie Weir impoundment is a low (<1 m high) gauging weir used to measure the water inflow.

### Sampling

To evaluate the fish community in the study area, fish sampling surveys were done over 17 months, between March 2020 and July 2021, during two low-flow and two high-flow periods. Five surveys were carried out: during 16–21 March 2020 at Spring Grove Dam impoundment (SGD); during 10–16 August 2020 at Inchbrakie Weir impoundment (IWP), downstream of Inchbrakie Weir (IWDS) and at Rosetta (RIV2); during 10–16 August 2020 at SGD, IWP, IWDS, RIV1 and Invermooi Estate (INVER); during 2–5 December 2020 at IWP, IWDS and INVER; and during 6–9 April 2021 and 2–8 July 2021 at all sites (Figure 1).

The sites were sampled using various methods. Fyke nets (4–8 nets of 19-mm mesh and opening height of 60 cm; T & L Netmaking, Mooroolbark, Australia) were deployed at each river site in the afternoon and checked every 12 h until they were removed at midday the next day. Four SGD sites were chosen to be representative, and a minimum of 12 gillnets (Eigevis Group of Companies, Cape Town, South Africa) were used per site overnight. At IWP, two gillnets were used overnight in the deepest section closest to the weir wall; the gillnets were 32 m long and 2 m high and incorporated a range of mesh sizes, from 16 mm to 150 mm (stretched mesh size). In addition, electrofishing (SAMUS 725M Electro-fisher, SAMUS Special Electronics, Poland) was conducted at all river sites that were wadable, as well as in wadable water at IWP.

All fish captured were measured to the nearest 5 mm standard length (SL) and then sorted into 50-mm size classes. Fish were identified following Skelton (2001). However, according to Weyl et al. (2017), then identification of two non-native bass species, *M. salmoides* and *M. floridanus*, is essentially impossible in the field and hybridisation occurs; even so, it is presumed that *M. salmoides* is present in the study area (Weyl et al. 2017).

Samples of surface water (1 000 ml) were collected at all sites and stored in a cooler box until being processed at Umgeni Water Laboratories (Pietermaritzburg). The water samples were analysed to determine whether water quality was in line with the guidelines of the Department of Water and Sanitation (previously the Department of Water Affairs and Forestry) and for use in the fish yield calculations (DWAF 1996).

### Data analyses

Data related to fish species diversity and abundance were analysed descriptively; data obtained from netting and electrofishing were used. We used the Kruskal−Wallis test to compare the size classes of fish caught between seasons (within habitat) and by habitat type (impoundment vs river), using the fyke net captures only. Kruskal−Wallis testing on non-parametric data has been used in size-class analysis for fisheries-related studies (Hlungwani et al. 2023). A histogram length-frequency plot was constructed to depict all fish that were caught across the study area. In addition, a histogram length-frequency plot was constructed specifically for captures in SGD to determine the population dynamics of fusiform fishes that would contribute to a commercial fishery venture; the African longfin eel *Anguilla mossambica* was excluded from this analysis as it is a catadromous species that moves out of fresh water to complete its life cycle.

All analyses pertaining to CPUE are descriptive because of the very low values obtained with the netting techniques and effort. For each netting technique, CPUE was calculated as CPUE = *C*_i_/*E*, where *C*_*i*_ is the total number of species *i* caught, and *E* is the effort performed in hours. The different netting techniques (i.e. fyke netting and gillnetting) were analysed separately.

No previous fisheries data were available for the Spring Grove Dam impoundment. Therefore, the potential yield was calculated using the formula developed by Marshall and Maes (1994) for African impoundments:

$$\text{Yield}(\frac{\text{kg}}{\text{ha}}\times \text{yr})=23.281\times {\left(\frac{\text{MD}}{\text{CON}}\right)}^{0.447}$$

where MD is the mean depth of the reservoir (m); and CON is conductivity, as measured during the sampling surveys and then averaged.

For comparative purposes, we also calculated potential yield using the global temperature-adapted morpho-edaphic index (MEI) model of Schlesinger and Regier (1982):

$$\text{Log}\;\text{Yield}(\frac{\text{kg}}{\text{ha}}\times \text{yr})=0.44\;\text{T}+0.482\mathrm{Log}\frac{\text{TDS}}{\text{MD}}+0.021$$

where T is the water temperature (°C), as measured during the fish sampling surveys and then averaged; TDS is total dissolved solids (mg l^−1^), as measured during the sampling surveys and then averaged; and MD is the mean depth of the reservoir (m).

All data analyses were conducted with R 4.1.1 (RStudioTeam 2021). A significance level of *p* < 0.5 was set for all statistical analyses.

## Results

### Species composition and distribution

A total of 147 fish representing nine species were caught and identified during the sampling surveys (Table 1; Figure 1). Only three were native fish species: the African longfin eel *Anguilla mossambica* (*n* = 4), chubbyhead barb *Enteromius anoplus* (*n* = 1) and KwaZulu-Natal yellowfish *Labeobarbus natalensis* (*n* = 5). The six non-native species were found in relatively high abundance: bluegill *Lepomis macrochirus* (*n* = 30), brown trout *Salmo trutta* (*n* = 51), rainbow trout *Oncorhynchus mykiss* (*n* = 11), spotted bass *Micropterus punctulatus* (*n* = 10), largemouth bass *M. salmoides* (*n* = 33) and smallmouth bass *M. dolomieu* (*n* = 2).

Fish abundance and diversity were the highest in SGD, with a total of 80 fish caught across the surveys, representing seven species (Table 1; Figure 1). Two species recorded at other sites were not recorded in the impoundment, namely *E. anoplus* and *M. dolomieu*. A total of 12 fish representing three species were caught at IWP: *A. mossambica* (*n* = 1), *M. salmoides* (*n* = 3) and *S. trutta* (*n* = 8). In terms of abundance, the most catches at a river site was for the most-upstream site, with 38 fish captured belonging to two species: *E. anoplus* (*n* = 1) and *S. trutta* (*n* = 37). The most-downstream site was dominated by two centrachid species: *M. salmoides* (*n* = 2) and *M. punctulatus* (*n* = 5). Ten fish were caught in the river stretch located between the two impoundments, which included the native *L. natalensis* (*n* = 1) and the non-native *M. dolomieu* (*n* = 2), *M. salmoides* (*n* = 2) and *S. trutta* (*n* = 5).

Ten species that were expected but not captured in the study area are: *Amphilius* natalensis, *Anguilla* bengalensis, *Clarias gariepinus, Ctenopharyngodon idella, Cyprinus carpio, Enteromius trimaculatus, E. viviparus, Labeo molybdinus, L. rubromaculatus* and *Oreochromis mossambicus*. Most of these species are on the edge of their distribution range, with *Cyprinus carpio* and *Ctenopharyngodon idella* being non-native species (Table 1).

### Size classes

**Figure 2:**
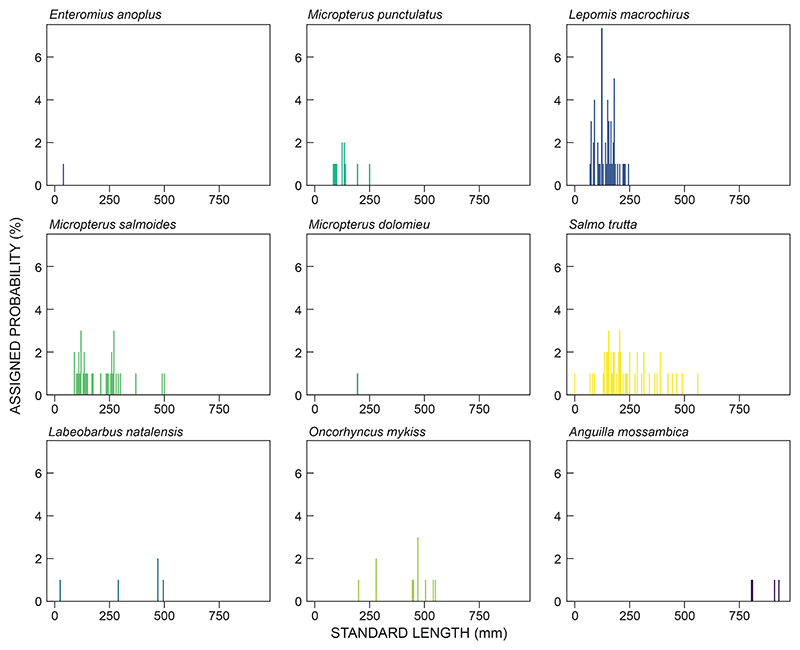
Length frequency plots for all fish species caught in the present study

**Figure 3:**
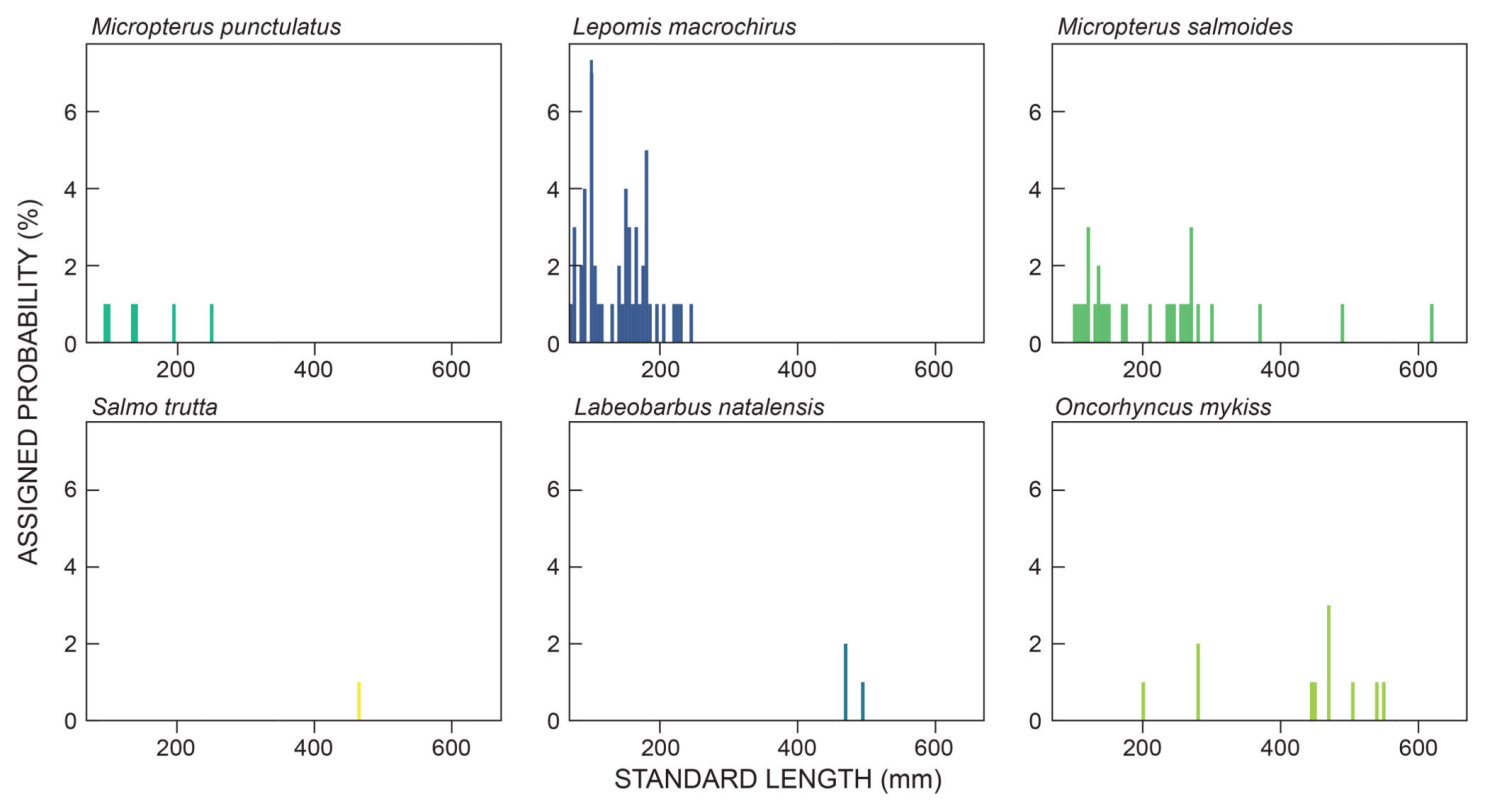
Length frequency plots for fusiform-shaped fish species caught in Spring Grove Dam, KwaZulu-Natal, South Africa, during the present study

Four species were caught at both river and impoundment sites: *Labeobarbus natalensis*, *M. punctulatus*, *M. salmoides* and *S. trutta* (Table 2; Figures 1−3). For these species, no significant differences in all size classes were observed between the two habitat types (Kruskal−Wallis, *p* = 0.327 for all species). Five species were caught during both low-flow and high-flow seasons: *Lepomis macrochirus, M. punctulatus, M. salmoides, O. mykiss* and *S. trutta*. No differences in sizes were observed between seasons for these species (Kruskal−Wallis, *p* = 0.6442 for all species). The size structure of the populations of *Lepomis macrochirus* and *M. salmoides* were well represented (Figures 2, 3).

### Calculated yield and CPUE

The yields for SGD were calculated using the calculated mean water depth (15.7 m), with the deepest observed point being 27 m. The dam wall height is 37 m, and when water levels are maintained between 50% and 60%, this reduces the deepest point in the dam, as seen throughout the study. Electrical conductivity did not exceed 60, with an average of 44.2 over the study period. These values were used in the formula of Marshall and Maes (1994) as follows: potential yield was calculated as 13.75 kg ha^−1^ annum^−1^, the surface area of Spring Grove Dam was estimated to be 1 022 ha (TCTA 2012), and tonne per year was calculated at 14.04 t yr^−1^.

Maximum TDS was 40 mg l^−1^, with a mean of 37.8 mg l^−1^; maximum water temperature was 20 °C, with an average of 15.8 °C. Using the means of TDS, temperature and depth (13.6 m), we calculated the Schlesinger and Regier (1982) MEI model as follows: potential yield was 7.18 kg ha^−1^ per annum and 7.34 t yr^−1^. Across all sites, habitat and season, values of CPUE were consistently low with both netting techniques for all species (Table 3). During the surveys, the dam’s water levels fluctuated between 60% and 100% of capacity.

Here, we also considered the netting techniques, as fyke nets and gillnets would be the only available methods for commercial fishers in the study area and this gear has been used in other studies in the region (Weyl et al. 2007; Barkhuizen et al. 2016). Our surveys combined 286 fishing efforts, totalling 3 961 h, yet yielded few fish. Fyke nets returned a CPUE with a maximum of 0.6 fish h^−1^ in the low-flow season, and 0.2 fish h^−1^ in the high-flow season. In total, 24 fishing efforts returned a CPUE that was not nil, against 91 unsuccessful efforts during the high-flow season. The results were similar during the low-flow season, with 19 fishing efforts yeilding a catch as compared with 79 efforts that failed to capture any fish. Among the successful fishing efforts using fyke nets, 25 were conducted in the impoundment and 18 in the river. We caught more species when using fyke nets (*n* = 8) than gillnets (*n* = 6), which included *A. mossambica, Labeobarbus natalensis, Lepomis macrochirus, M. dolomieu, M. punctulatus, M. salmoides, O. mykiss* and *S. trutta*.

**Figure 4:**
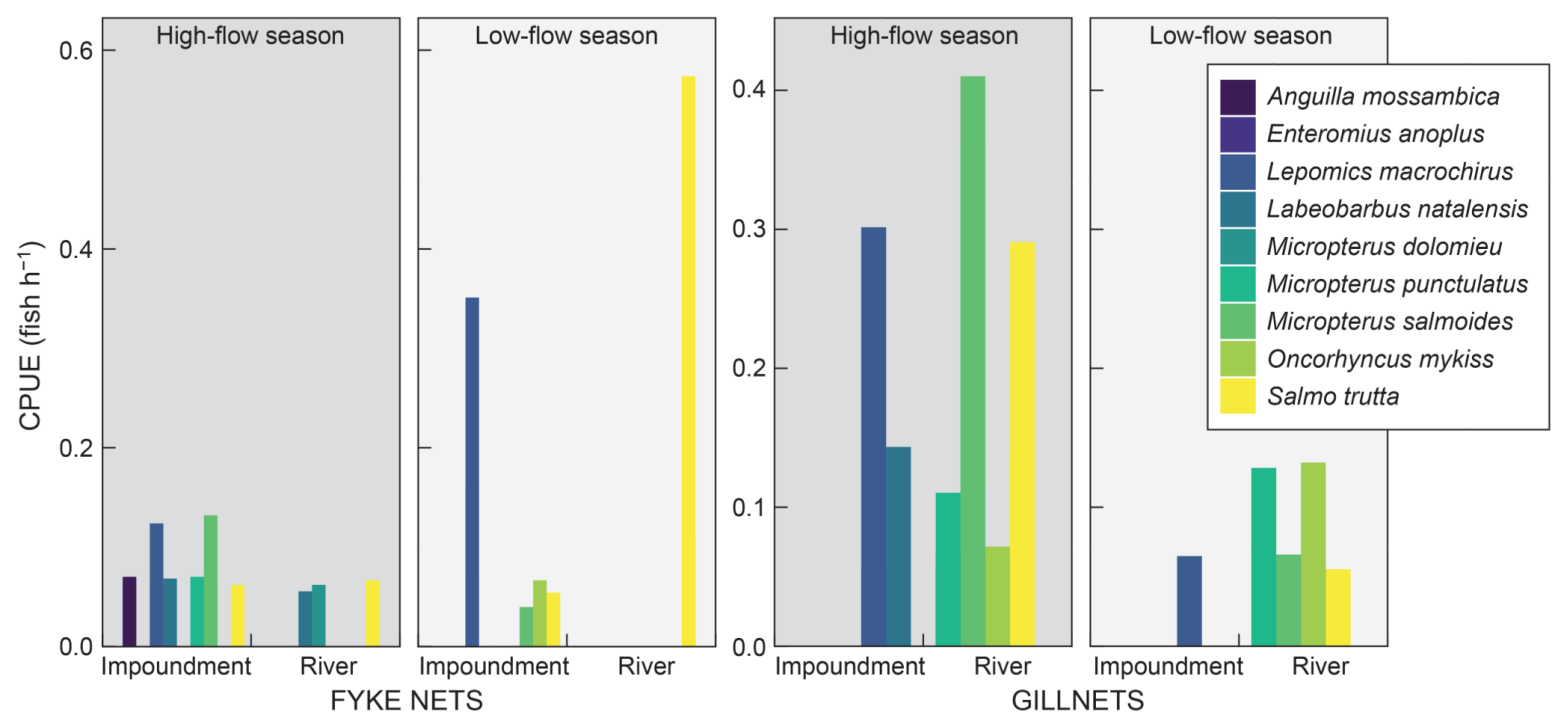
Maximum catch per unit effort (CPUE) with the different netting techniques used in this study, by season and habitat

Gillnets returned a CPUE of a maximum 0.7 fish h^−1^ in the high-flow season and a maximum of 0.2 fish h^−1^ in the low-flow season (Figure 4). The high-flow season might be more suitable for this technique, as the most effort occurred in this season. A total of 14 fishing efforts were successful using gillnets in the high-flow season (mean CPUE 0.2 fish h^−1^), compared with 9 efforts in the low-flow season (mean CPUE 0.1 fish h^−1^). The six species caught using gillnets were *Lepomis macrochirus, Labeobarbus natalensis, M. puctulatus, M. salmoides, O. mykiss* and *S. trutta*.

## Discussion

The present study is the first comprehensive investigation of the fish community in the recently constructed Spring Grove Dam. The dam’s construction severely altered the river habitats and caused the inundation of Inchbrakie Falls, creating conditions favouring non-native fishes and the proliferation of centrarchids. This has led to their establishment in the dam in under 8 years. Inchbrakie Weir was constructed to mitigate the reservoir’s inundation of Inchbrakie Falls and to prevent the upstream movement of *M. dolomieu* (Figure 1). The subsequent challenges and ecologicial issues created at this location are similar to those at many other impoundments used as water storage facilities across South Africa and abroad. Shortcomings as an impoundment for inland fisheries are low CPUE, low yields and poor commercial fishery management (Weyl et al. 2007; Barkhuizen et al. 2016; Abobi and Wolff 2020; Camp et al. 2020). Furthermore, impoundments invariably negatively impact aquatic ecosystems (Mantel et al. 2017; O’Brien et al. 2019) such as by providing altered habitats more suitable for non-native and invasive species (Barkhuizen et al. 2017). Managing the small-scale fisheries in the study area will require biodiversity consideration, given the NEM:BA and NFEPA significance assigned to this location (Nel et al. 2011; DEFF 2021).

### Fish community

The fish community recorded in the present study consists primarily of non-native species, specifically salmonids and centrarchids, with only three native species caught. The low abundance of the expected native species can be related to poor ecological connectivity, altered flow regimes, as well as the relatively high abundances of non-native species, as seen in other studies (Evans et al. 2022). This is concerning when managing biodiversity endpoints and promoting native fisheries.

The endemic *L. natalensis* is a popular angling fish and grows to sizes comparable to many salmonids (Crass 1964; Karssing 2008; Burnett et al. 2021). High-elevation rivers (>1 000 m a.s.l.), similar to in the study area, would be considered the upper altitudinal limit of their distribution range, which is limited by cooler temperatures and migratory access between winter and summer habitats (Crass 1964; Burnett et al. 2021). *Labeobarbus natalensis* at times exhibits dormancy during low temperatures in high altitudinal reaches (Crass 1964), where it is also vulnerable to predation by salmonids (Crass 1964). The introduction of *S. trutta* shifted the distribution of *L. natalensis* from its preferred cooler extralimital temperature range to warmer downstream waters (Crass 1964). Despite this, there can be a niche overlap between *S. trutta* and *L. natalensis*, with their co-existence observed in some mid-altitudinal waters in KZN (Harrison 1950; Crass 1964; Evans et al. 2022). This niche overlap could shift in response to climate changes, as warming waters at high altitudes are expected (Rivers-Moore et al. 2019), reducing the extent of suitable habitat for salmonids (Kovach et al. 2016, 2019).

Anguillids were previously considered pests and were killed when caught (Harrison 1950; Harrison et al. 1963), as they were thought to reduce the abundance of trout (Turnbull-Kemp 1958; Butler and Marshall 1996). However, a worrying decline in their range distribution has been observed in KZN (Pike et al. 2020; Hanzen et al. 2022). Their low abundance in the present study likely implicates impaired river connectivity in the catchment caused by artificial instream barriers. Anguillid eels are long-distance migrators requiring connectivity between the sea and rivers to complete their life cycle (Hanzen et al. 2019, 2022). International interest in the trade of African anguillid eels is growing (Gollock et al. 2018) as temperate anguillid populations cannot sustain commercial demands (Jacoby et al. 2015; Righton et al. 2021). An evaluation of eel fisheries and demand in South Africa is much needed (Hanzen et al. 2019). Since the construction of Spring Grove Dam, there is a concern that eels will no longer be recruited into the area, limiting the promotion of these species in inland fisheries, barring interventions to aid their natural recruitment.

*Enteromius anoplus* belongs to the species complex of chubbyhead barbs and was possibly stocked in the region as a fodder food for salmonids; however, little public information or records of their stocking localities and abundance were ever kept (Johnson et al. 2009; Cox and Mather 2018). Translocating fish allows for gene mixing, even while a given species is still recognised (Impson et al. 2008). In the Eastern Cape, a population previously recognised as *E. anoplus* has now been described as a separate species, *E. mandelii*; this suggests that species variation in the *E. anoplus* complex may be linked to specific catchments (Kambikambi et al. 2021). This could be the case for other species like *L. natalensis* (Stobie et al. 2018). *Enteromius anoplus* are likely threatened by *S. trutta* because trout are known to impact the abundance of minnows *Enteromius* spp. (Gratwicke and Marshall 2001; van der Walt et al. 2019). In our sampling surveys, no *E. anoplus* were caught where centrarchids were present, including downstream of Spring Grove Dam. Similarly, despite its high-elevation distribution, *A. natalensis* was not detected in the present study (Marriot et al. 1997; Skelton 2001; Karssing et al. 2012). Other studies highlight that *A. natalensis* and *E. anoplus* populations are under threat from predatory pressure by non-native species (Woodford et al. 2005; Jackson et al. 2016b), with downgraded assesments by the International Union for Conservation of Nature (IUCN) (Chakona et al. 2022).

The lack of expected native species in the present study is concerning, even if the finding is not surprising since the study area falls within their known range limits (Evans et al. 2022). Many of these species, such as *C. gariepinus* and *Labeo rubromaculatus*, are seasonal migrators moving upstream in summer and downstream in winter (Skelton 2001). The presence of artificial barriers downstream of the study area, and Spring Grove Dam has subsequently restricted upstream seasonal movements, limiting their range to below these instream barriers (Mantel et al. 2017; Evans et al. 2022). The relatively high altitude and cooler temperatures associated with the study area are not conducive for the expected subtropical species to survive through the winter without expressing some form of dormancy or access to migration routes, as seen in *Labeobarbus natalensis* (Crass 1964; Burnett et al. 2021). Similar to the African anguillids, consideration for the migration of these species is neglected when building large storage dams (Mantel et al. 2017; O’Brien et al. 2019) like Spring Grove Dam, with no fishway nor eelway included.

Both centrarchids and salmonids can have a negative impact on native aquatic biodiversity in South Africa, especially fish diversity (Gratwicke and Marshall 2001; Wasserman et al. 2011; Karssing et al. 2012; Rivers-Moore et al. 2013, 2019). Mitigating their impact in the study area was the intended purpose of the Inchbrakie Weir. Centrarchids were most abundant in the present study, primarily downstream of Inchbrakie Weir, which has primarily kept invasive centrarchids out of the upper reaches of the Mooi River. However, *M. salmoides* was detected in the weir’s impoundment; its presence might be attributable to dispersal downstream from nearby farm dams rather than from below the weir (Nemai 2006; TCTA 2012), and its persistence can be explained by the presence of a favourable lotic habitat created by the impoundment (Alexander et al. 2014; Khosa et al. 2019). Notably, a gauging weir at the inlet to Inchbrakie Weir potentially serves as an additional barrier against *M. salmoides* moving upstream into waters already occupied by *S. trutta*. Historically, *S. trutta* were stocked in the river as hatchery-reared fry. This historical investment (Natal Parks Board 1974) supported the construction of Inchbrakie Weir as a fish barrier to protect the trout-related economy from the invasion of migrating *M. dolomieu* after Inchbrakie Falls was inundated owing to construction of the dam (Skelton 2000a; TCTA 2011). The impact of *M. dolomieu* on aquatic fauna has led to a marked reduction in fish species abundance (Woodford et al. 2005). A few studies have shown that *S. trutta* and *M. dolomieu* negatively impact each other because they tend to occupy different niches (Brown 2004; Cucherousset and Olden 2011) and have little overlap in their non-native and native distributions. At this stage, the impact of *M. salmoides* on the trout fishery in the present study and their upstream movements are unknown. Even so, this species should be monitored given potential economic fallouts to local stakeholders.

### Catch rates and yields

Based on the fish community structures, the CPUE and the calculated yield for Spring Grove Dam in the present study, the viability of the impoundment for small-scale commercial fisheries is not supported. Low yields can be attributed to low electrical conductivity originating from cool headwater streams. Expected yields for other high-elevation dams in KZN, such as Spioenkop, Chelmsford and Woodstock, were estimated to exceed 400 t yr^−1^ (Britz et al. 2015); however, this has never been obtained, and presently these impoundments do not sustain commercial fisheries. Midmar Dam, with a surface area of 1 540 ha, had no calculated potential yield (Britz et al. 2015) despite interest by the Department of Water and Sanitation in developing commercial fisheries there. Historic estimated commercial output and recommended yield quotas were unrealistic and unobtainable, resulting in failed commercial fisheries (Barkhuizen et al. 2017). When comparing other studies that used similar yield calculations following Marshall and Maes (1994) and Schlesinger and Regier (1982), Weyl et al. (2007) showed that impoundments relatively similar in size to Spring Grove Dam had higher yields. These are likely because of warmer temperatures, higher conductivity levels, and higher abundance and diversity of native fishes, which can support better production (Weyl et al. 2007; Barkhuizen et al. 2016). Of the ten impoundments that Weyl et al. (2007) surveyed in South Africa, only one was recommended for commercial fishing, reaching an MEI yield of 187*–*291 t yr^−1^. In other, West African impoundments, calculated values of the MEI showed considerably higher yields per surface area than that in the present study though similar to that reported by Weyl et al. (2007) (Abobi and Wolff 2021). Fish yield calculations across Africa using models are sparse, primarily from the lack of data required for the model calculations (Tesfaye and Getahun 2021). The MEI yields do not account for variability in a dam’s capacity, while such variability was observed in Spring Grove Dam, and is a recognised research gap in fisheries management (Weyl et al. 2021). The fluctuations in water volume stem from water transfer between catchments as needed to meet urban water demands, and this needs to be considered if fisheries are to be an added benefit in the construction of a water storage facility (Weyl et al. 2021), especially in more drought-prone areas. During the present study, the Spring Grove Dam impoundment seldom reached full capacity, with large and frequent water-level fluctuations experienced. Such patterns can potentially negatively impact fish abundance and population dynamics, and could be a reason for low CPUE. However, this does not help to explain the high abundances of non-native centrarchids in Spring Grove Dam.

Weyl et al. (2007) mention that several factors limit commercial fisheries, not just yields. Notably, the distance to high-density human communities and the ease of access to the market also impose limitations (Weyl et al. 2007; Barkhuizen et al. 2016, 2017). These limitations are present for Spring Grove Dam in addition to the low yield calculated in the present study. There are little data available for comparison in South Africa, particularly for high-elevation dams, largely because of failed reporting rates to the FAO, reduced national interest in inland fisheries, and frequent failure of commercial fishing ventures (McCafferty et al. 2012; Britz et al. 2015; Barkhuizen et al. 2016).

Compared with the estimates of CPUE, similar results were found for potential yields, that is the low CPUE values aligned with the poor calculated yield for Spring Grove Dam. This might be attributable to low productivity associated with a high elevation, low electrical conductivity, and cooler water temperatures which are characteristic across the Drakensberg Mountain Range (Evans et al. 2022). Values of CPUE in impoundments in a subtropical region also yielded higher abundances in the study by Weyl et al. (2007) than in the present study. Impoundments constituting small water storage facilities at high elevations (Barkhuizen et al. 2017) displayed CPUE values similar to those in the present study. Overall, this highlights the importance of calculating yields for each dam on a case-by-case basis rather than applying estimates across a region (Weyl et al. 2007). The existence of low CPUE-associated impoundments in such areas supports the need to reconsider the sustainability of commercial fisheries in dams constructed as a water storage facility (Barkhuizen et al. 2017). A preferred approach could be to promote subsistence fishers through small-scale ventures and maintain continual interest in recreational fisheries (Barkhuizen et al. 2017).

### Fisheries management recommendations for Spring Grove Dam

Considering that small-scale commercial fisheries are not viable at Spring Grove Dam, we recommend that subsistence fishing (including small-scale fisheries) and continued recreational fisheries specific to certain species should be supported. Five species of interest in developing the recreational fisheries are *S. trutta, O. mykiss, M. salmoides, M. dolomieu* and *L. natalensis*, all caught in the present study.

Our results show that the Spring Grove Dam impoundment can support subsistence fishing (including small-scale commercial) and a recreational fishery focused on *Micropterus* spp. Importantly, this would apply to the impoundment only; preventing the spread of *M. dolomieu* upstream of Inchbrakie Weir must be maintained. The presence of *M. salmoides* upstream of this weir would conflict with stakeholders’ interest in maintaining the recreational salmonid fishery. Furthermore, as non-native fishes, a range expansion of *Micropterus* spp. should be twarted to protect fish diversity. The construction of Inchbrakie Wier as a fish barrier seems to have prevented the expansion of *M. dolomieu* and can be used as a barrier to eliminate *M. salmoides* upstream through an adequate eradication programme (Weyl et al. 2014; van der Walt et al. 2019; Dalu et al. 2020). This does not address the presence of salmonids upstream of the fish barrier and their impact on aquatic biodiversity. Since Spring Grove Dam has become operational, the task of eradicating invasive species (especially downstream of Inchbrakie Weir) is costly, highlighting the economic importance of restricting the movement of non-native species outside of their present distribution ranges.

Selective harvesting of centrarchids should be encouraged to reduce their abundance, either through recreational fishing or bass-fishing tournaments, and can support subsistence fishers. A creative and potentially viable option would be the promotion of Spring Grove Dam as a bass spearfishing venue, as the water clarity would allow for this (Boshoff 2019). Reducing the centrarchid population would enhance the present multispecies recreational and subsistence fisheries, as fishing pressure alone would not completely eradicate the centrarchid species.

The management of non-native species is likely to be affected by the NEM:BA-listed alien invasive species, as gazetted in 2021, pending the listing of salmonids (Van Wilgen and Wilson 2018; DEFF 2021). The implematation of NEM:BA has been under continual legal opposition from lobbyists who argue that the Act threatens the livelihoods of those dependent on the salmonid fishery (Ellender et al. 2014). Despite this, there is a need to revisit the 1974 Game and Fisheries Ordinances (Natal Parks Board 1974) to device a holistic approach to fisheries management in KZN, which will include native fish species that have been overseen or neglected or are tied to thriving fisheries communities (e.g. *Labeobarbus* spp., clarids and cichlids). This need is presently acknowledged (pers. comm., EKZN Wildlife). Decision support tools are available to balance the non-native versus native species conundrum in fisheries management, as developed by Kimberg et al. (2014) and Woodford et al. (2017), which could also be used for Spring Grove Dam. We suggest that the section of the Mooi River upstream of Inchbrakie Weir remains an area to benefit the salmonid resources, with the exception of protected areas such as the Kamberg Nature Reserve (Maloti*–*Drakensberg Park World Heritage Site), where native biodiversity, especially fish diversity, should be prioritised and even mandated over any non-native fishery (Weyl et al. 2015).

Salmonids hold important economic value and can only be fished for in waters at higher elevations in the region, including in the study area (Du Preez and Lee 2010; Weyl and Cowley 2015). This allows an important salmonid recreational fishery to exist upstream of Inchbrakie Weir, where the populations and abundances of trout species supported this. Keeping bass *Micropterus* spp. out of this reach is important to maintain the trout fishery. However, consideration must be given to the influence of a changing climate which portends the decline of salmonids (Rivers-Moore et al. 2007, 2019). Provisionally, according to the NEM:BA lists, a permit would be required to stock a salmonid species in private waters or raise them in hatcheries (DEFF 2020, 2021). This does not differ much from the present KZN ordinances (cf. Natal Parks Board 1974). There is clear ecological evidence that salmonids have invasive potential (Karssing et al. 2012; Rivers-Moore et al. 2013, 2019; Ellender et al. 2014; Jackson et al. 2016b) and accordingly an invasive label is often assigned to them (Marr et al. 2017; Weyl et al. 2020).

Emphasis should be given to a catch-and-release recreational fishery for the native *L. natalensis*, owing to the low numbers found in the present study as well as the interest in recreational fishing for members of this genus (Impson et al. 2008). Species of *Labeobarbus* throughout their distribution have a historical association with subsistence fishers (Skelton 2000b; Mitchell et al. 2012), but until their abundances improve in Spring Grove Dam (Supplementary Information S1), subsistence fishing for *L. natalensis* should be ruled out. Moreover, the presence of *L. natalensis* increases native fish diversity (DEFF 2021).

Enhancing the fishery for *L. natalensis* could be considered for the study area, especially for the sake of fish diversity (see recommended fishing regulations in Supplementary Information S2). Careful management of the centrarchid species is needed to ensure minimal damage to the native aquatic fauna and the established *S. trutta* population, especially upstream of Inchbrakie Weir. Therefore, methods to remove centrarchids from the in Spring Grove Dam impoundment should be investigated to reduce species’ competition, protect existing populations and possibly improve the recreational fisheries for salmonids and *L. natalensis*. Conservation of the genetic resources of native fishes requires consideration in fishery management. Fish genetic resources can be compromised by inter-basin water transfer schemes, like the Mooi*–*Mgeni Transfer Scheme in the present study (Nemai 2006), as they allow fish to move into adjacent water courses, thereby impacting gene flow (Ellender and Weyl 2014; Harris et al. 2022). Inter-basin transfer schemes can move potentially invasive native species into new waters or genetically mix variations within species. Presently, *L. natalensis* is considered one species but with several regional variations (Crass 1964) that may be elevated to species level (Stobie et al. 2018). Conservation authorities and water resource managers need to mitigate against hybridisation (Impson et al. 2008). An engineered solution is needed to prevent fish from moving with water pumped through the dam. This would allow *L. natalensis* as well as non-native species to be included in any fishery in Spring Grove Dam without jeopardising genetic conservation (see potential management strategies for *L. natalensis* in Supplementary Information S1 and S2). Conclusive research on *L. natalensis* is still needed to guide decisions on its genetic management, as there seemed little consideration for this aspect of fishery management when constructing then Spring Grove Dam. Restricting fish through the pumping scheme will also reduce the spread of non-native species.

## Conclusions

Spring Grove Dam fisheries management depends mainly on agreements between stakeholders and local governing authorities. Commercial fisheries in this impoundment are unreliable. Instead, subsistence, small-scale fisheries based on obtained yields for invasive non-native species can be considered. Such fisheries would not conflict with the established recreational fisheries, which should implement a no-take principle for native fish species. All catch data should be recorded and centralised for stakeholders, as actual fishing records can improve yield calculations and future management decisions. In addition, the management of subsistence and recreational fisheries should be evaluated seasonally using these data. A long-term objective guided by an updated fishery evaluation and the growing complexities around NEM:BA could achieve a well-managed fishery for salmonids and *L. natalensis*, given clear uptake from stakeholders and holistic fishery directives for the area. To achieve this, management measures must be taken to mitigate the range expansion of centrarchids or even to eradicate them in the impoundment. Concerning climate-change predictions, promoting *L. natalensis* as the main targeted fish in the study area would be beneficial once clarity around their genetic conservation is obtained. Regulating fishing practices for *L. natalensis* can ensure successful recruitment and growth of the population, as was obtained for trout via the 1974 ordinances (Natal Parks Board 1974) (Supplementary Information S1 and S2); uptake for this would have to come from the angling community.

The lack of a fisheries monitoring programme is concerning, as any future recommendations for the fisheries associated with Spring Grove Dam need these data to guide management. Monitoring and further research on the aquatic fauna can aid in understanding the impact of non-native species and can improve fishery management in the study area. The present study provides some baseline data and calculated yields for consideration; however, long-term data would validate our recommendations and feed into an adaptive management plan.

The poor resilience of native species as a consequence of the negative impacts of habitat inundation and the proliferation of non-native species is concerning, especially in waters where centrarchids are present. The long-term survival of freshwater anguillids upstream of Spring Grove Dam needs to be addressed to improve their conservation status, especially in terms of planned dam developments on South Africa’s east-flowing rivers. Water storage facilities and the instream barriers they create increase the abundance of invasive species, alter the river landscape and reduce native species, making biodiversity, especially fish diversity, difficult to manage. Finally, water storage facilities should be only cautiously promoted for their potential as commercial fisheries. Instead, the investment in maintaining economies around the existing subsistence and recreational fisheries, especially for native species, should be prioritised.

## Supplementary Material

Supplementary material: available online at https://doi.org/10.2989/16085914.2024.2373096

Supplementary Information S1;S2 and Supplementary Table S1

## Figures and Tables

**Table 1:** List of expected fish species in the Mooi River catchment, KwaZulu-Natal, South Africa, as per the Freshwater Biodiversity Information System (FBIS, freshwaterbiodiversity.org), and catches of the species recorded in the present study. Conservation status as per the International Union for Conservation of Nature (IUCN): VU = Vulnerable; NT = Near Threatened; LC = Least Concern

Expected species	Present study (*n*)	Origin	Conservation status
*Salmo trutta*	51	Non-native	–
*Micropterus salmoides*	33	Non-native	–
*Lepomis macrochirus*	30	Non-native	–
*Oncorhynchus mykiss*	11	Non-native	–
*Micropterus punctulatus*	10	Non-native	–
*Labeobarbus natalensis*	5	Native	LC
*Anguilla mossambica*	4	Native	NT
*Micropterus dolomieu*	2	Non-native	LC
*Enteromius anoplus*	1	Native	LC
*Amphilius natalensi*s	0	Native	VU
*Anguilla bengalensis*	0	Native	NT*
*Clarias gariepinus*	0	Native	LC
*Ctenopharyngodon idella*	0	Non-native	–
*Cyrpinus carpio*	0	Non-native	–
*Enteromius trimaculatus*	0	Native	LC
*Enteromius viviparus*	0	Native	LC
*Labeo molybdinus*	0	Native	LC
*Labeo rubromaculatus*	0	Native	VU
*Oreochromis mossambicus*	0	Native	VU

* Least Concern for Africa

**Table 2:** Summary of the sizes of the fish species caught in the present study. SL = standard length

Species	SL min. (mm)	SL max. (mm)	SL mean (mm)
*Anguilla mossambica*	805	930	863.7
*Enteromius anoplus*	40	40	40.0
*Lepomis macrochirus*	71	245	136.9
*Labeobarbus natalensis*	24	495	349.8
*Micropterus dolomieu*	195	195	195.0
*Micropterus punctulatus*	85	250	128.2
*Micropterus salmoides*	90	620	204.1
*Oncorhynchus mykiss*	200	550	423.6
*Salmo trutta*	70	560	234.6

**Table 3:** Catch per unit effort (CPUE, number of fish h^−1^) for all fish species caught with all capture techniques during the sampling surveys

Species	CPUE max.	CPUE mean
*Anguila mossambica*	0.070	0.001
*Lepomis macrochirus*	0.351	0.006
*Labeobarbus natalensis*	0.144	0.001
*Micropterus dolomieu*	0.062	0.000
*Micropterus punctulatus*	0.128	0.002
*Micropterus salmoides*	0.410	0.007
*Oncorhyncus mykiss*	0.132	0.002
*Salmo trutta*	0.574	0.008

## Data Availability

The data belong to the University of KwaZulu-Natal and are available from the lead author on reasonable request.
