# Supplementary material for: Potential for a commercial inland fishery or just another water storage facility at Spring Grove Dam, KwaZulu-Natal, South Africa?
Source: Afr J Aquat Sci. Author manuscript; Available in PMC 2025 Dec 11. (PMC7618466; doi:10.2989/16085914.2024.2373096)
Supplement: Supplementary Information S1;S2 and Supplementary Table S1 [file EMS211450-supplement-Supplementary_Information_S1_S2_and_Supplementary_Table_S1.pdf]

SUPPLEMENTARY MATERIAL

Potential for a commercial inland fishery or just another water storage facility at Spring Grove Dam, KwaZulu-Natal, South Africa?

Matthew J Burnett, Céline Hanzen, Alex Whitehead, Gordon C O'Brien and Colleen T Downs

African Journal of Aquatic Science 49(2): 145–158
https://doi.org/10.2989/16085914.2024.2373096

Supplementary Information S1: Potential management of the *Labeobarbus natalensis* fishery at Spring Grove Dam and in relation to Inchbrakie Fish Barrier, KwaZulu-Natal, South Africa (see main text for a list of references)

A possibility with the growing interest in *Labeobarbus natalensis* would be to establish a population upstream of the Inchbrakie Weir. These species rely on pools, and have been observed to use impoundments as refugia in the cooler winters (Burnett et al. 2021), which is potentially provided for by the Inchbrakie Wier impoundment. Introducing *L. natalensis* upstream of Inchbrakie Weir would conflict with the already established *Salmo trutta* fishery, but not as severely as the already present *Micropterus salmoides*. This may allow a gradual shift from a salmonid fishery to a native-based *L. natalensis* fishery as climate change predicts increases in temperature for the region (Rivers-Moore et al. 2007). This will see extensive limitations to the distribution of salmonids, and will potentially threaten the economy around *Salmo trutta* (Rivers-Moore et al. 2007, 2019). Therefore, the potential to provide an indigenous alternative merits future consideration. Salmonids and *L. natalensis* do not presently co-exist in the upper Bushman and Mngeni river systems (Evans et al. 2022) but are likely in the upper Mooi River. However, further biological and ecological research, as well as a market evaluation, would need to be conducted by stakeholders, conservation bodies and governing bodies to show the feasibility of such a venture. Historical records of *L. natalensis* are sparse for the section upstream of Inchbrakie Weir, especially before the salmonid introductions. However, climate change predictions and the tolerance of *L. natalensis* to cooler waters elsewhere suggest that the species could occur upstream of Inchbrakie Falls (Crass 1964; Burnett et al. 2021). The observation of *L. natalensis*, in particular collection of a juvenile specimen, downstream of Inchbrakie Weir indicate that this fish is capable of breeding in these reaches while using the Spring Grove Dam impoundment to endure harsh winters.

Supplementary information S2: Proposed recreational angling regulations for Spring Grove Dam impoundment to Inchbrakie Weir, KwaZulu-Natal Province, South Africa (see main text for a list of references)

The Mooi River below Inchbrakie Weir to the Spring Grove Dam impoundment is included as part of Spring Grove Dam (see Figure in main text). These proposed regulations are under the present conditions, although if management decisions are taken to enhance the fisheries, these may change. Below are regulatory recommendations and suggestions for the targeted angling species (see Supplementary Table S1):

- For *Labeobarbus natalensis*, the bag limits from the 1974 ordinances need to be re-evaluated, particularly across KwaZulu-Natal as a whole, with specific regulations per catchment and water body. For the present study area, a strict catch-and-release rule should be imposed because abundance is low and this native species is threatened by alien invasive species.
- To protect the salmonid fishery, similar bag limits set in 1974 could be acceptable. However, re-evaluation of the ordinances is needed to align with protecting native fish species that can be sensitive to angling pressure. The proposed regulations follow previous ordinances and set a limit of 10 fish per bag and fish must be at least 25 cm in fork length.
- An unlimited bag limit is recommended for centrarchids for both up and downstream of Inchbrakie Weir and in the Spring Grove Dam impoundment, with active removal practice to reduce abundances. The invasiveness of centrarchids will not be deterred by fishing pressure, for various reasons, as anglers target large specimens and practice catch and release that is hard to discourage (Carey et al. 2015). Fishing pressure for non-native invasive fishes is not known to eradicate populations but does reduce abundances, which can alleviate pressure on native fish populations (Barfoot et al. 2002; Carey et al. 2015). Non-native invasive fish eradication programmes require more drastic measures than relying on recreational or sports angling pressure alone (van der Walt 2019). The only successful invasive fish eradication programmes in South Africa have been conducted using rotenone, a registered pesticide, under favourable conditions, such as strategically placing it instream barriers to prevent recolonisation by the fish (van Der Walt et al. 2019; Dalu et al. 2020).

Supplementary Table S1: Tabulated recommendations for fish species at Spring Grove Dam, KwaZulu-Natal, South Africa, based on fish records in the present study. The regulations are proposed with the exclusion of species on the NEM:BA list (National Environmental Management – Biodiversity Act 10 of 2004: RSA [2004]), which are marked here with an asterisk (*), and include current KwaZulu-Natal regulations from 1974, which need to be re-evaluated

Species	Spring Grove Dam impoundment	Inchbrakie Weir pool (IWP)	Upstream of IWP
Anguilla mossambica	Not to be targeted; must be released if caught	Not to be targeted; must be released if caught	Not to be targeted; must be released if caught
Enteromius anoplus	Not to be targeted; must be released if caught	Not to be targeted; must be released if caught	Not to be targeted; must be released if caught
Lepomis macrochirus	No bag limit on number or size	May not be released into these waters	May not be released into these waters
Labeobarbus natalensis	Strictly catch and release; wading on spawning beds prohibited in the river from 1 October to 31 April	N/A	N/A
Micropterus dolomieu	No bag limit on number or size	If caught notify authorities. May not be released into these waters	If caught notify authorities. May not be released into these waters
Micropterus punctulatus	No bag limit on number or size	May not be released into these waters	May not be released into these waters
Micropterus salmoides	No bag limit on number or size	Removed when caught, must not be released into these waters	May not be released into these waters
*Oncorhynchus mykiss**	No more than 10 fish per bag limit, and fish cannot be <25 cm in length	No more than 10 fish per bag limit, and fish cannot be <25 cm in length	No more than 10 fish per bag limit, and fish cannot be <25 cm in length
*Salmo trutta**	No more than 10 fish per bag limit, and fish cannot be <25 cm in length. Waters closed from 16 May through 31 August each year	No more than 10 fish per bag limit, and fish cannot be <25 cm in length. Waters closed from 16 May through 31 August each year	No more than 10 fish per bag limit, and fish cannot be <25 cm in length. Waters closed from 16 May through 31 August each year
